# DAB2IP loss confers the resistance of prostate cancer to androgen deprivation therapy through activating STAT3 and inhibiting apoptosis

**DOI:** 10.1038/cddis.2015.289

**Published:** 2015-10-29

**Authors:** J Zhou, Z Ning, B Wang, E-J Yun, T Zhang, R-C Pong, L Fazli, M Gleave, J Zeng, J Fan, X Wang, L Li, J-T Hsieh, D He, K Wu

**Affiliations:** 1 Department of Urology, The First Affiliated Hospital of Xi'an Jiaotong University, Xi'an, Shaanxi, China; 2 Department of Urology, University of Texas Southwestern Medical Center, Dallas, TX, USA; 3 Vancouver Prostate Center, University of British Columbia, Vancouver, British Columbia, Canada

## Abstract

Loss of *DAB2IP*, a novel tumor suppressor gene, is associated with the high risk of aggressive prostate cancer (PCa). Previously, we reported that DAB2IP modulated androgen receptor activation in the development of castration-resistant PCa; however, its direct action on the failure of androgen deprivation therapy (ADT) remains largely unknown. In this study, we showed that DAB2IP knockdown could significantly enhance *in vitro* growth and colony formation of PCa cells following ADT as well as tumorigenicity in pre-castrated nude mice. In addition, DAB2IP loss stabilized mitochondrial transmembrane potential, prevented release of cytochrome *c*, Omi/HtrA2 and Smac from the mitochondria to the cytoplasm and inhibited intrinsic apoptosis induced by ADT. Mechanistically, DAB2IP could interact with the signal transducer and activator of transcription 3 (STAT3) via its unique PR domain and suppress STAT3 phosphorylation and transactivation, leading to the inhibition of survivin expression in PCa cells. Moreover, the luminal epithelia in DAB2IP^−/−^ mice with more activated STAT3 and survivin expression were resistant to castration-induced apoptosis. Consistently, DAB2IP expression inversely correlated with STAT3 phosphorylation and survivin expression in PCa patients. Together, our data indicate that DAB2IP loss reprograms intracellular signal transduction and anti-apoptotic gene expression, which potentiates PCa cell survival from ADT-induced cell death.

Prostate cancer (PCa) is the most prevalent cancer and the second leading cause of cancer deaths in men in the United States.^1^ Although surgery or radiotherapy can effectively control the primary PCa, 30% patients have already developed metastatic lesions at diagnosis.^2^ Androgen deprivation therapy (ADT) has become a standard therapy for metastatic PCa, because PCa is an androgen-dependent disease. Unfortunately, PCa recurs eventually and develops to be the lethal castration-resistant PCa (CRPC).^3^ ADT failure leading to castration resistance is a multi-factorial process by which cells acquire survival advantage in the absence of androgens and proliferate using non-androgenic factors.^4, 5^ Elucidating these pathways is a critical step towards identifying potential target and developing new therapeutic strategies.

From searching genomic-wide database, a single nucleotide polymorphism probe from *DAB2IP* gene, a new member of the RAS-GTPase-activating protein family,^6^ is associated with the high risk of aggressive PCa.^7^ Downregulation of *DAB2IP* gene expression, mainly due to epigenetically silencing,^8^ is correlated with advanced PCa with high tumor grade^9, 10, 11^ and increases distant metastasis or the resistance to radio- and chemotherapy in PCa.^10, 12, 13^ In recent times, we also demonstrate that DAB2IP loss is associated with hyperactivation of androgen receptor (AR), indicating its critical roles in the development of CRPC; however, the direct evidence for DAB2IP dysregulation leading to the failure of ADT is still lacking.

In this study we unveil that DAB2IP loss accelerates the androgen-independent outgrowth through protecting PCa cells from apoptotic cell death induced by ADT. Mechanistically, DAB2IP could bind to the signal transducer and activator of transcription 3 (STAT3) and modulated its phosphorylation and transactivation, and then reprogrammed a subset of anti-apoptotic or pro-apoptotic gene expression (i.e., survivin, Bcl-2 and Bax). Subsequently, DAB2IP loss could prevent ADT-induced alteration of mitochondrial membrane potential, release of cytochrome *c*, Omi/HtrA2 and Smac from the mitochondria to the cytoplasm and Caspase cascade leading to apoptosis. Together, we conclude DAB2IP/STAT3/survivin as a critical pathway leading to the survival advantage of PCa cells during ADT treatment.

## Results

### DAB2IP loss potentiates androgen-independent growth and colony formation of PCa cells after ADT

**Figure 1:**
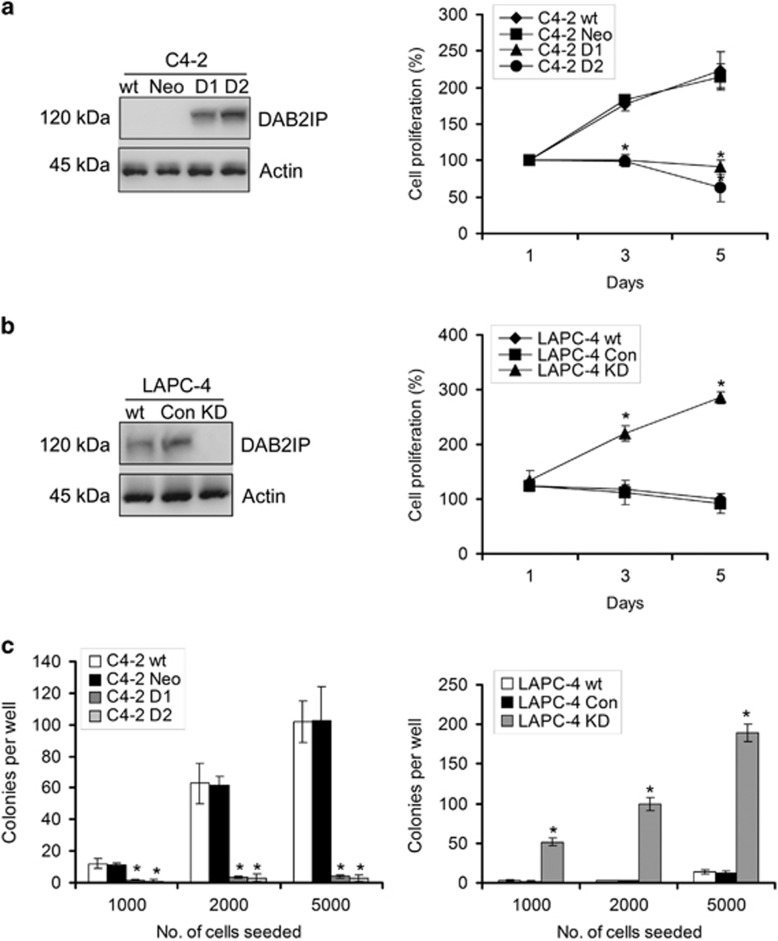
DAB2IP modulates the cell survival of PCa cells under androgen-depleted condition. (**a**–**c**) C4-2 and LAPC-4 sublines were cultured in Phenol Red-free RPMI-1640+5% CS-FBS and cell growth was determined by MTT or colony formation assay; data (means±S.E.M.) were obtained from three independent experiments; *P<0.05. Ectopic or endogenous DAB2IP expression in PCa sublines was confirmed by western blotting

DAB2IP is known to suppress androgen-elicited PCa growth;^11^ herein, we further examine whether DAB2IP could alter the androgen-independent growth under androgen-depleted condition. As shown in Figure 1a, the presence of DAB2IP in androgen-independent C4-2 cells (i.e., D1 and D2) significantly suppressed cell growth in Phenol Red-free medium with charcoal-stripped FBS (CS-FBS; *P*<0.05). In contrast, knockdown of DAB2IP in androgen-responsive LAPC-4 (i.e., KD) cells exhibited an accelerated cell growth compared with parental LAPC-4 or vector control (i.e., Con) cells (*P*<0.05; Figure 1b). Consistently, we found that the D1 and D2 cells formed fewer colonies compared with parental C4-2 or Neo cells (*P*<0.05; Figure 1c (left panel) and Supplementary Figure S1A), whereas LAPC-4 KD cells maintained higher colony-formation capacity than both LAPC-4 wild-type (wt) or Con cells under androgen-depleted condition (*P*<0.05; Figure 1c (right panel) and Supplementary Figure S1B).

We further determined *in vivo* tumor take rate of these cells in pre-castrated nude mice. As shown in Table 1, data from subcutaneous xenograft model indicated that C4-2 Neo cells could form tumors 10 days after injection and overall tumor take rate was 55.6% (4 weeks) and 88.9% (8 weeks), but D2 cells could not form any detectable tumors within this period. Similar results were also observed in LAPC-4 cells. Only two of nine mice developed subcutaneous tumors in LAPC-4 Con cells (take rate 22.2%); in contrast, the tumor take rate of LAPC-4 KD cells was 77.8% 8 weeks after injection (*P*<0.001; Table 1). All these data indicate that DAB2IP loss in PCa cells could maintain tumor growth after ADT.

### DAB2IP controls the intrinsic apoptosis of PCa cells following ADT

**Figure 2:**
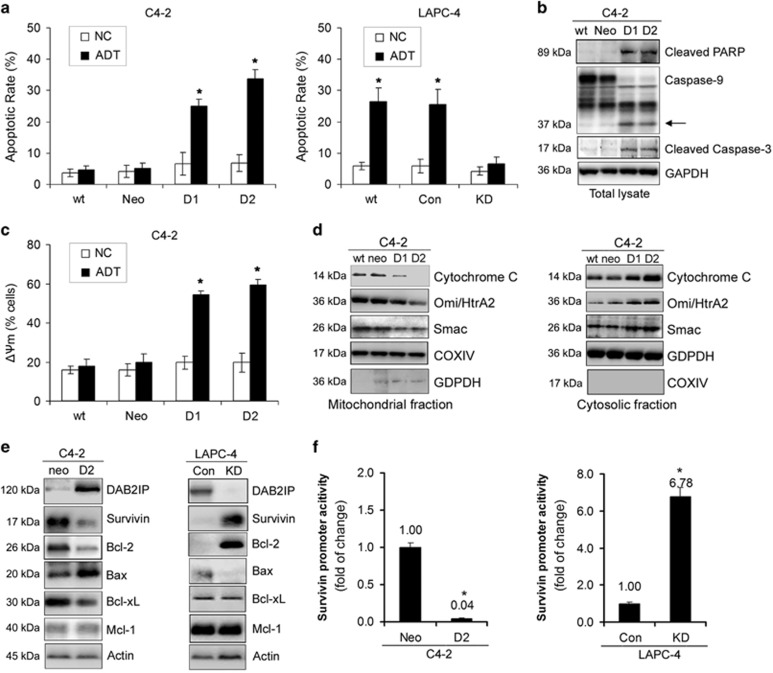
DAB2IP controls the intrinsic apoptosis of PCa cells following ADT. PCa sublines were cultured in Phenol Red-free RPMI-1640+5% CS-FBS for 3 days. (**a**) C4-2 (left) and LAPC-4 (right) sublines were then collected for Annexin V/FITC staining and flow cytometric analysis, and data (means±S.E.M.) were obtained from three independent experiments; *P<0.05. (**b**) Total lysates from C4-2 sublines were prepared to analyze cleaved PARP, Caspase-9 and cleaved Caspase-3 by western blotting; GAPDH was used as a total loading control. (**c**) C4-2 sublines were collected for JC-1 staining and flow cytometric analysis, and data (means±S.E.M.) were obtained from three independent experiments; *P<0.05. (**d**) Mitochondrial (left) or cytosolic (right) fractions from C4-2 sublines were prepared to analyze cytochrome *c*, Omi/HtrA2 and Smac by western blotting, COX IV and GAPDH were used as mitochondrial or cytosolic loading controls, respectively. (**e**) Cell lysates of C4-2 and LAPC-4 sublines were extracted for western blotting, to detect DAB2IP, survivin, Bcl-2, Bax, Bcl-xL and Mcl-1. Actin was used as loading control. (**f**) C4-2 and LAPC-4 sublines were transiently transfected with survivin pLuc-230 for 48 h along with the pRL-SV40 *Renilla* luciferase construct as internal control and then the promoter activity of survivin was determined by Dual-Luciferase Reporter Assay. Each result was performed in triplicate. *P<0.05

It is known that ADT causes apoptosis of androgen-dependent prostatic epithelia cells^14^ and the presence of DAB2IP enhances apoptosis of PCa cells after inhibition of AKT activity.^15^ We therefore examine whether DAB2IP has effects on ADT-induced apoptosis. Indeed, as measured by flow cytometry the percentage of apoptotic cells was significantly higher in C4-2 D1 and D2 cells compared with wt or Neo cells (*P*< 0.05; Figure 2a, left panel), whereas fewer LAPC-4 KD cells underwent apoptosis than wt and Con cells under androgen-depleted condition (*P*<0.05; Figure 2a, right panel). Consistently, the elevated cleaved subunits of Caspase-3 (17 kDa), Caspase-9 (17 kDa) and PARP (89 kDa) were detected in C4-2 D1 and D2 cells following ADT (Figure 2b). Mitochondrial disruption is considered as an early step in triggering intrinsic apoptosis.^16^ We also observed that ADT significantly induced the loss of mitochondrial membrane potential (ΔΨm) in C4-2 D1 and D2 cells compared with wt or Neo cells (*P*<0.05; Figure 2c) and increased the release of cytochrome *c*, Omi/HtrA2 and Smac from the mitochondria to the cytoplasm (Figure 2d). All these data indicate that DAB2IP could destabilize mitochondrial membrane by releasing apoptogenic molecules to the cytoplasm and activating Caspase cascade.

### DAB2IP regulates the expression of pro-apoptotic and anti-apoptotic genes

To dissect the possible mechanism of DAB2IP in mitochondrial disruption, we surveyed gene expression profile using cDNA microarray and found that the expression of multiple pro-apoptotic and anti-apoptotic genes including *Bax*, *Bid*, *Bad*, *Bak1*, *Bcl-2*, *Bcl-xL*, *Mcl-1*, *survivin* and *livin* was differentially regulated by DAB2IP in C4-2 and LAPC-4 cells (Supplementary Table S1). Indeed, western blot analyses confirmed that C4-2 D2 cells exhibited higher expression of pro-apoptotic genes (i.e., *Bax*) but lower expression of anti-apoptotic genes (i.e., *survivin*, *Bcl-2* and *Bcl-xL*) compared with Neo cells. In contrast, LAPC-4 KD cells with endogenous DAB2IP knockdown expressed higher levels of anti-apoptotic genes (i.e., *survivin* and *Bcl-2*) but lower levels of pro-apoptotic genes (i.e., *Bax*) compared with Con cells (Figure 2e). In addition, luciferase assay further demonstrated that DAB2IP could significantly modulate survivin promoter activity in these cells (Figure 2f).

### DAB2IP interacts with STAT3 via its PR domain to suppress STAT3 phosphorylation and transactivation

**Figure 3:**
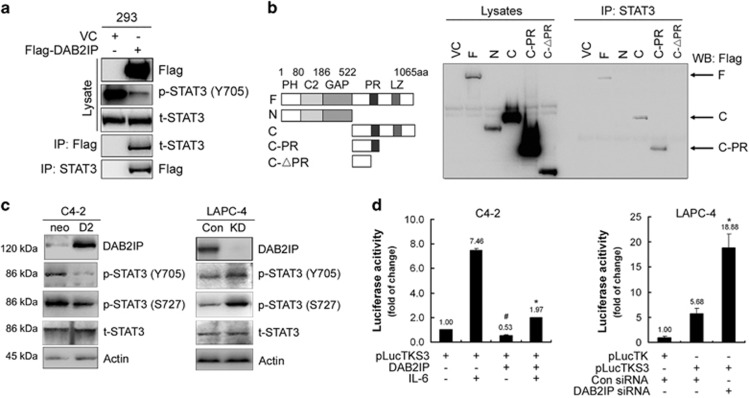
DAB2IP interacts with STAT3 and inhibits its activation in PCa cells. (**a**) HEK 293 cells were transfected with Flag-DAB2IP and cell lysates were subjected to co-immunoprecipitation probed with Flag and then probed with t-STAT3 antibody, or subjected to co-immunoprecipitation probed with t-STAT3 and then probed with Flag antibody. (**b**) Left panel: schematic depiction of PR domain mutant constructs; right panel: *in vivo* analysis of binding domain in DAB2IP to STAT3. After transfecting with DAB2IP-F, N, C, C-PR, C-ΔPR cDNA constructs, HEK 293 cells were subjected to immunoprecipitation with t-STAT3 and then probed with Flag antibody. (**c**) Cell lysates of C4-2 and LAPC-4 sublines were extracted for western blotting, to detect DAB2IP, p-STAT (Y705), p-STAT3 (S727) and t-STAT3. Actin was used as loading control. (**d**) Left panel: C4-2 cells were co-transfected with STAT3-responsive luciferase reporter pLucTKS3 and DAB2IP or vector control for 24 h and then treated with IL-6 (10 ng/ml) for another 24 h. The luciferase activity was measured using the Dual-Luciferase Reporter Assay System. Right panel: LAPC-4 cells were co-transfected with pLucTKS3 or its control pLucTK and DAB2IP siRNA or scramble siRNA for 48 h before collecting for luciferase assay. Each result was performed in triplicate. *P<0.05

Survivin is one of the key members of inhibitor of apoptosis (IAP) gene family, which has been implicated in the resistance to antiandrogen therapy in PCa.^17^ Therefore, we explored the mechanism of DAB2IP in regulating *survivin* gene transcription. Based on a screening using the mass spectrometry (Supplementary Table S2) and co-immunoprecipitation, we showed that the PR domain of DAB2IP could directly bind to STAT3 protein (Figures 3a and b, and Supplementary Figure S2). Subsequently, lower expression of phosphorylated STAT3 (p-STAT3) at tyrosine 705 (Y705) and serine 727 (S727) was detected in C4-2 D2 cells than in Neo cells, whereas higher levels of p-STAT3 (Y705) and p-STAT3 (S727) were detected in LAPC-4 KD cells than in Con cells (Figure 3c). Similar results were also observed in other PCa cells (i.e., PC-3) or immortalized human normal-prostate epithelial cells (i.e. PZ-HPV-7 and RWPE-1) after DAB2IP knockdown (Supplementary Figure S3). In addition, immunohistochemistical (IHC) staining data showed that LAPC-4 KD xenograft tissues exhibited significantly elevated p-STAT3 (Y705) and survivin expression compared with Con tissues (Supplementary Figure S4).

Furthermore, using a specific STAT3-responsive luciferase reporter we showed that DAB2IP could significantly suppress both baseline and induction of STAT3 transactivation elicited by interleukin-6 (IL-6) treatment in C4-2 cells, meanwhile LAPC-4 KD cells showed a significantly increased baseline of STAT3 transcription activity (Figure 3d). Moreover, similar data were also observed in other PCa cell lines, such as LNCaP, DU145 and PC-3 (Supplementary Figure S5), indicating a universal regulation of STAT3 transactivation by DAB2IP in PCa cells.

### STAT3/survivin mediated the resistance of DAB2IP-deficient PCa cells to apoptosis induced by ADT

**Figure 4:**
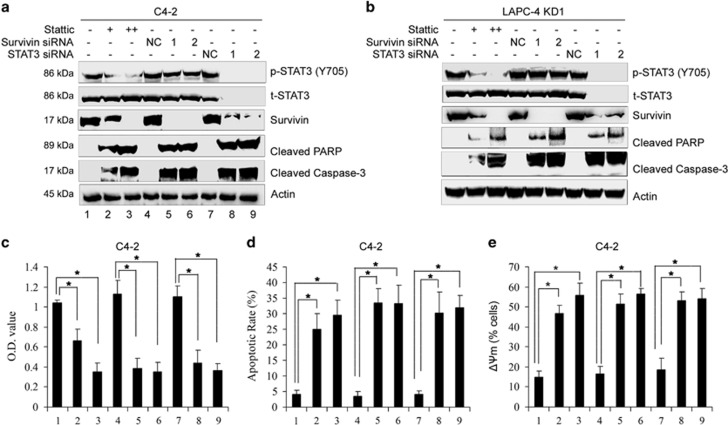
STAT3/survivin signaling pathway mediates the resistance to ADT-induced apoptosis in DAB2IP-deficient PCa. C4-2 or LAPC-4 KD cells were pretreated with specific STAT3 inhibitor Stattic (10 or 20 *μ*M) for 1 h or transfected with siRNA oligonucleotides specific to STAT3 or survivin for 24 h, and then cultured in Phenol Red-free RPMI-1640+5% CS-FBS for another 48 h. (**a** and **b**) Cell lysates after treatment were subjected to western blotting for detecting p-STAT3 (Y705), t-STAT3, survivin, cleaved PARP and Caspase-3. Actin was used as a loading control (**c**) C4-2 cells after treatment were cultured in Phenol Red-free RPMI-1640+5% CS-FBS for another 48 h and cell growth was determined by MTT assay. (**d**) C4-2 cells after treatment were cultured in Phenol Red-free RPMI-1640+5% CS-FBS for another 48 h, then subjected to Annexin V/FITC staining and flow cytometric analysis. (**e**) C4-2 cells after treatment were cultured in Phenol Red-free RPMI-1640+5% CS-FBS for another 48 h, then subjected to JC-1 staining and flow cytometric analysis. All data (means±S.E.M.) shown in **c**, **d** and **e** were obtained from three independent experiments. *P<0.05 between indicated groups

To further investigate the role of STAT3 signalling in regulating survivin expression and cell apoptosis, we targeted STAT3 with either siRNA or specific inhibitor Stattic.^18^ The expression of survivin was inhibited in DAB2IP-deficient C4-2 or LAPC-4 KD cells by STAT3 siRNA or inhibitor (Figures 4a and b), indicating that activated STAT3 signaling is crucial for survivin expression. Consistently, knockdown or inhibition of STAT3 and survivin significantly inhibited cell growth, increased cell apoptosis and induced the loss of ΔΨm in C4-2 cells following ADT (Figures 4c–e). Notably, western blot analysis also showed that knockdown or inhibition of STAT3 and survivin led to cell apoptosis evidenced by the elevation of cleaved Caspase-3 and PARP (Figures 4a and b). In addition, we showed that overexpression of survivin or constitutively active STAT3C could abolish the effects of DAB2IP on the induction of cell apoptosis in C4-2 D2 cells under the same condition based on western blot analysis (Supplementary Figure S6). All these data indicate that STAT3/survivin axis mediates the resistance of DAB2IP-deficient PCa cells to ADT-induced apoptosis.

### Prostate epithelia from DAB2IP^−/−^ mice exhibited active STAT3/survivin signaling and resistance to castration-induced apoptosis

**Figure 5:**
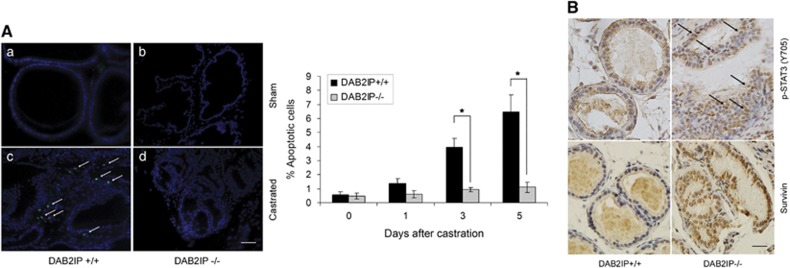
Prostate epithelia from DAB2IP^−/−^ mice maintain active STAT3/survivin signaling and resistance to castration-induced apoptosis. (**A**) DAB2IP^+/+^ or DAB2IP^−/−^ mice were castrated for indicated time and then killed to collect prostate. *In situ* apoptosis was detected by TUNEL assay (identified by green FITC staining). Left panel: representative pictures were shown with or without castration treatment (**a**–**d**); right panel: quantitative data for percentage of TUNEL-positive apoptotic cells in prostate tissues with or without castration treatment. Data (mean±S.E.M.) were generated from five different mice in each group; *P<0.05. (**B**) Prostate tissues from male DAB2IP^+/+^ and DAB2IP^−/−^ mice were collected and then subjected to IHC staining with p-STAT3 (Y705) or survivin antibodies. Arrowhead indicates STAT3 nuclear location in the mouse prostatic epithelium. Representative pictures of IHC staining were shown. Scale bar=50 *μ*m

Furthermore, we used knockout mouse model to confirm our observation *in vitro*. Using terminal deoxynucleotidyl transferase-mediated dUTP nick end labeling (TUNEL) staining to measure the apoptotic index of mouse prostatic epithelia following castration, the level of epithelial apoptosis in DAB2IP^+/+^ mice gradually increased and peaked at Day 5; however, no significant increase of the TUNEL-positive cells was detected in the prostate gland of DAB2IP^−/−^ mice at the same time, suggesting that DAB2IP is a key effector in castration-elicited apoptosis in prostatic epithelia *in vivo* (Figure 5A). To be consistent, more nuclear staining of p-STAT3 (Y705) and survivin was observed in the epithelial compartment of prostate gland from DAB2IP^−/−^ mice (Figure 5B).

### DAB2IP loss correlates with p-STAT3 and survivin expression in clinical specimens

**Figure 6:**
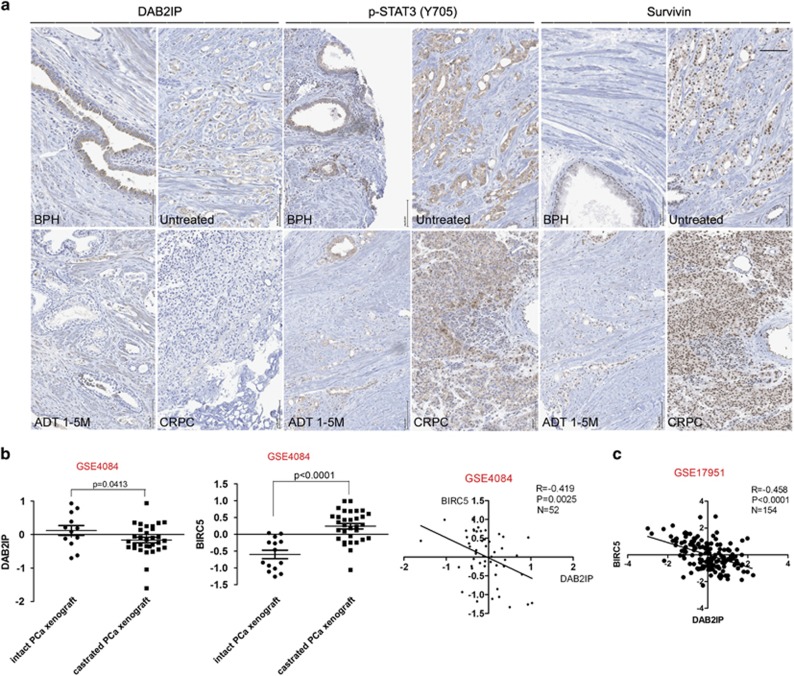
Correlation of DAB2IP, p-STAT3 and survivin expression in clinical PCa or PDX tissues. (**a**) Representative IHC staining of DAB2IP, p-STAT3 (Y705) and survivin in clinical specimens (BPH, untreated, ADT 1-5 month (M) and CRPC) was shown. Scale bar=50 *μ*m. (**b**) Microarray data set (GSE4084) with 52 human PCa xenografts was retrieved from the NCBI GEO and log2 data for individual probes was *Z* scored for plotting. Left and middle panel: relative expressions of DAB2IP and survivin (encoded by the *BIRC5* gene) in intact or castrated mice were shown; right panel: Spearman's correlation coefficient of DAB2IP and survivin expression was shown. (**c**) Microarray data set (GSE17951) with 154 human PCa tissues was retrieved from the NCBI GEO and log2 data for individual probes was *Z* scored for plotting. Spearman's correlation coefficient between DAB2IP and survivin was shown

We also conducted IHC staining in PCa tissue microarrays (TMAs) to detect the expression of survivin and p-STAT3 (Y705), and analysed their correlations with DAB2IP expression level reported in our previous studies.^11^ Indeed, we found that higher survivin expression was uniquely detected in high-grade or CPRC tissues and no survivin was detected in benign prostate tissues (Figure 6a), indicating its unique role as a proto-oncogene. Noticeably, the expression of survivin decreased soon after ADT, then restored to the higher status in CRPC (Figure 6b and Supplementary Figure S7); a similar pattern was observed with p-STAT3 expression (Figure 6a and Supplementary Figure S8). Overall, an inverse correlation of survivin or p-STAT3 expression with DAB2IP staining was detected in TMAs containing 276 PCa tissues: Pearson's correlation coefficient is −0.76 between survivin and DAB2IP, and −0.45 between p-STAT3 (Y705) and DAB2IP. In addition, NCBI GEO database (GSE4084) containing the gene profiles of human patient-derived xenografts (PDXs) collected from male mice up to 14 days after castration also showed a significantly increased expression of survivin but decreased expression of DAB2IP in the castrated mice (Figure 6b, left and middle panel); indeed, there is an inverse correlation between DAB2IP and survivin expression in these mouse xenografts or other human PCa tissues (GSE17951) (Figure 6b (right panel) and Figure 6c). Overall, these data strengthen the notion that DAB2IP loss could unleash STAT3 activation, leading to survivin gene expression, which is expected to contribute to ADT resistance or CRPC development.

## Discussion

The current treatment for metastatic PCa is medical or surgical castration and ADT is the most effective regimen. Unfortunately, patients ultimately relapse and develop CRPC associated with the mortality of this disease. Unveiling the mechanisms that underlie PCa castration-resistant progression after ADT will facilitate the development of novel effective therapies. Previous studies have identified DAB2IP as an inhibitor of epithelial-to-mesenchymal transition, leading to PCa metastases.^9, 12^ In this study we clearly show that DAB2IP loss also potentiates PCa androgen-independent growth *in vitro* and *in vivo*.

It is well known that PCa depends on a crucial level of androgenic stimulation for growth and survival. ADT causes cancer regression, because without androgen the rate of cell proliferation is lower and the rate of apoptosis increased, leading to extinction of these cells.^19^ We conclude that DAB2IP can modulate STAT3 activation and reprograms its target gene expression (i.e., *survivin*, *Bcl-2* and *Bax*), which will shift the balance from pro-apoptotic to anti-apoptotic activities under androgen-depleted condition. In fact, DAB2IP not only induces cell apoptosis but also reduces cell proliferation of PCa cells, which have been demonstrated in our previous study.^15^

Among various pro-apoptotic events, the disruption of ΔΨm and release of cytochrome *c* from the mitochondria to the cytoplasm are considered critical in mitochondria-mediated intrinsic apoptosis pathways.^20^ In this study we showed a dissipation of ΔΨm in DAB2IP-overexpressing PCa cells as evidenced by JC-1 staining, which was accompanied by the release of cytochrome *c*, Omi/HtrA2 and Smac from the mitochondria to the cytoplasm and further activation of downstream effectors Caspase-9, Caspase-3 and PARP. All these data indicate that DAB2IP inhibits intrinsic apoptosis induced by ADT.

A recent study from Di Minin *et al.*^21^ has first discovered that p53 mutants could interact with DAB2IP and induce a TNF-dependent transcriptional profile via NF-*κ*B and JNK, which was crucial for the invasive phenotype of cancer cells exposed to inflammation. Herein, we show that DAB2IP regulates the expression of these anti-apoptotic or pro-apoptotic genes, which could also be transcriptionally regulated by p53. Therefore, it is interesting to investigate the p53 status in our cell models. However, we did not find any correlation between DAB2IP and mutant p53 expression in prostate cells (Supplementary Figure S9).

Accumulating evidence has demonstrated that aberrant AR signaling has contributed to the androgen-independent growth of PCa;^22^ however, bypass pathways will provide a substitute survival signal to PCa cells when crucial AR signaling is targeted by ADT. Blocking the apoptosis signal would be one such pathway for tumor cell survival.^17, 23^ Indeed, our previous study has shown that DAB2IP can modulate AR activities and PCa cell growth.^11^ In addition to AR inhibition, this study further demonstrates that DAB2IP can modulate androgen-independent pathway that is involved in PCa cell survival after ADT.

Evidence for an essential role of activated STAT3 in preventing the apoptosis of human tumor cells was first shown in multiple myeloma.^24^ STAT3 activation supports tumor cell survival by upregulating expression of the anti-apoptotic proteins (i.e., Bcl-2, Bcl-xL, Mcl-1 and survivin), cyclin D1 and Myc.^18, 25, 26, 27^ In general, STAT3 is constitutively activated in diverse cancers. Many tumor-produced factors, such as IL-10, IL-6 and VEGF, activate STAT3 by efficient feed-forward mechanisms. STAT3 and its associated factors (i.e., IL-6) have been shown to have a crucial role in pro-survival and growth of PCa cells in androgen-depleted condition.^28, 29, 30^ Our data clearly show that DAB2IP could inhibit STAT3 activity through dephosphorylation of its two residues, tyrosine 705 and serine 727, both of which are critical for STAT3 dimerization and transactivation,^31, 32^ and then selectively control the expression of these target genes (i.e., *survivin* and *Bcl-2*) *in vitro* and *in vivo*. It is consistent with previous studies that survivin mediates resistance to ADT in PCa,^17^ whereas Bcl-2 expression is augmented following ADT and is correlated with androgen-independent progression of PCa.^23^ In addition, Min *et al.*^9^ have also reported similar results that showed DAB2IP knocking down PrEC cells expressed higher levels of *IL-6*, *VEGF*, *Bcl-2*, *Bcl-xL*, *C-IAP1*, *C-IAP2* and *Myc* genes, while DAB2IP-overexpressing PC-3 cells expressed lower levels of *IL-6*, *C-IAP1*, *C-IAP2* and *Myc* genes, and all these genes were consistent with the activation status of STAT3. Furthermore, the interaction of the unique PR domain of DAB2IP with STAT3 may have a critical role, because our previous observation has shown that the PR domain in DAB2IP functions as a new inhibitory class of PR domain in modulating PI3K-Akt and Src-AR activity.^11, 15^ Taken together, these data provide supporting evidence that DAB2IP loss facilitates PCa cell survival after ADT through activating STAT3.

In summary, this study delineates additional novel molecular mechanism of DAB2IP in modulating cell survival and apoptosis, which appears to be the key pathway leading to ADT resistance. As both STAT3 and AR activities are aberrantly elevated and have crucial roles in CRPC,^33, 34^ we believe that dual-targeting therapeutic strategy based on the unique molecular structure of DAB2IP may provide a potential opportunity to treat CRPC.

## Materials and Methods

### Cells and reagents

Stable DAB2IP-expressing sublines (i.e., D1, D2) of androgen-independent C4-2 with its empty vector control (i.e., Neo) subline and stable DAB2IP-knockdown subline (i.e., KD) of androgen-responsive LAPC-4 with its empty vector control (i.e., Con) subline were generated previously.^12^ C4-2 cells were maintained in T-medium (Gibco, San Diego, CA, USA) containing 5% (v/v) FBS (Invitrogen, Carlsbad, CA, USA) and LAPC-4 cells were maintained in Iscove's modified Dulbecco's medium (Gibco) containing 10% FBS at 37 °C with 5% CO_2_ in a humidified incubator. To deplete androgen, cells were sub-cultured in Phenol Red-free RPMI-1640 with 5% or 10% CS-FBS (Hyclone, Omaha, NE, USA). Antibodies for p-STAT3 (Tyr705), p-STAT3 (Ser727), Bcl-2, Bcl-xL, Mcl-1, Caspase-9, cleaved Caspase-3 and cleaved PARP were purchased form Cell Signaling Biotechnology (Beverly, MA, USA). Antibodies for t-STAT3, survivin, Bax, p53, cytochrome *c*, Omi/HtrA2, Smac, GAPDH and COX IV were purchased from Santa Cruz Biotechnology (Santa Cruz, CA, USA). Antibody for Actin was purchased form Sigma-Aldrich (St. Louis, MO, USA).

### siRNA oligonucleotides, plasmids and cell transfection

Two different pairs of STAT3 or survivin-specific siRNA oligonucleotides and control siRNA oligonucleotides were designed and purchased from GenePharma (Shanghai, China). The sequence of STAT3 or survivin siRNAs used in this study was as follows: STAT3 siRNA-1 sense 5′-CACCGCAUCUCUACAUUCATT-3′ and antisense 5′-UGAAUGUAGAGAUGCGGUGTT-3′ STAT3 siRNA-2 sense 5′-CUGAGAACGAGCCAGACUUTT-3′ and antisense 5′-AAGUCUGGCUCGUUCUCAGTT-3′ survivin siRNA-1 sense 5′-GGGACCUGGUGUGAAUUAUTT-3′ and antisense 5′-AUAAUUCACACCAGGUCCCTT-3′ survivin siRNA-2 sense 5′-CCCGGAAAUUUAACAUUCUTT-3′ and antisense 5′-AGAAUGUUAAAUUUCCGGGTT-3′ Control siRNA sense 5′-GCACCACUUCCAGGGUUUATT-3′ and antisense 5′-UAAACCCUGGAAGUGGUGCTT-3′. Specific siRNA oligonucleotides for human DAB2IP and control siRNA were previously described.^15^ Survivin cDNA and constitutely active STAT3C plasmids were kindly provided by Dr Allen Gao (University of California at Davis, Sacramento, CA, USA).^35^ Transfections were performed using Lipofectamine RNAiMax (Invitrogen) according to the manufacturer's protocol.

### Clinical specimens

The prostate TMAs including 276 tissue specimens obtained from Vancouver Prostate Centre, University of British Columbia, were commonly used in our previous studies.^10, 11, 36^ The Institutional Review Board approved the tissue procurement protocol in this study and appropriate informed consent was obtained from all patients.

### MTT assay

Cell growth rate was determined by MTT (3-(4,5-dimethylthiazol-2-yl)-2,5-diphenyltetrazolium bromide) assay as previously described.^11^ Briefly, cells were seeded into Phenol Red-free RPMI-1640 with CS-FBS at the density of 5 × 10^3^ cells/well in 96-well plates for different time indicated before MTT assay (Roche, Indianapolis, IN, USA). The absorbance (OD) was measured at 590 nm using the SpectraMax microplate reader (Molecular Devices, Sunnyvale, CA, USA). Independent experiments were repeated in triplicates.

### Colony formation assay

A total of 1000, 2000 or 5000 cells per well were seeded in 6-well plates for 24 h and then switched into Phenol Red-free RPMI-1640 medium containing 5% or 10% CS-FBS for 2 weeks, and fresh medium was changed every 3–4 days. The plates were then washed with ice-cold PBS, fixed with 4% paraformaldehyde, stained in crystal violet solution for 15 min at room temperature and washed with distilled water to remove excess dye. The number of colony was counted for each sample.

### Apoptosis and mitochondrial membrane potential analyses

Cells were treated and cultured in Phenol Red-free RPMI-1640 containing CS-FBS for 3 days and then collected and washed with PBS. Annexin V/FITC staining was performed with a kit from Invitrogen. JC-1 staining was performed with a kit from Cayman Chemical Company (Ann Arbor, MI, USA). Cell apoptosis and mitochondrial membrane potential (ΔΨm) were determined by flow cytometric analysis (FACSCalibur, BD Biosciences, Franklin Lakes, NJ, USA). Independent experiments were repeated in triplicates.

### Preparation of mitochondrial and cytosolic fractions

Mitochondrial and cytosolic extracts of treated cells were prepared as previously described.^20^ Briefly, cells were incubated with permeabilization buffer (250 mmol/l sucrose, 20 mmol/l HEPES/KOH (pH 7.4), 1 mmol/l EGTA, 1 mmol/l EDTA, 1 mmol/l DTT, 0.1 mmol/l PMSF, 1 mg/ml chymostatin, 1 mg/ml leupeptin, 1 mg/ml antiparin and 1 mg/ml pepstatin A). Cells were homogenized and centrifuged at 500 × *g* for 10 min, to pelletize the nucleus and cell debris. The supernatants were further centrifuged at 13 000 × *g* for 30 min. Cytosolic supernatants and mitochondrial pellets were collected. The pellets were then suspended in mitochondrial lysis buffer (150 mmol/l NaCl, 50 mmol/l Tris-HCl (pH 7.4), 1% NP-40, 0.25% sodium deoxycholate, 1 mmol/l EGTA and protease inhibitor). After vigorous vortex and centrifugation at 15 000 × *g* for 15 min, the mitochondrial fraction was collected. All the samples were stored at −80 °C until use.

### Immunoprecipitation and western blot analysis

HEK 293 cells were transfected with different DAB2IP expression vectors. For immunoprecipitation, transfected HEK 293, RWPE-1 or LAPC-4 cells were washed twice with cold PBS and lysed in 1.5 ml of cold lysis buffer for 20 min on ice. The immunocomplex was subjected to western blot analysis as described previously.^11^

### Luciferase reporter gene assay

For the reporter gene assay, cells seeded in 24-well plates were transfected with 200 ng STAT3-responsive luciferase reporter plasmid pLucTKS3 or the control plasmid pLucTK^37^ and 1 ng of the pRL-SV40 *Renilla* luciferase construct (as an internal control). Cell extracts were prepared 24 h after incubation with a STAT3-activated cytokine IL-6 (10 ng/ml) and the luciferase activity was measured using the Dual-Luciferase Reporter Assay System (Promega).

### RNA isolation and cDNA microarray

Total cellular RNA was extracted from C4-2 and LAPC-4 sublines with RNeasy Plus Mini kit (Qiagen) and then subjected to microarray analysis. All RNA labeling and cDNA microarray preparations were performed by The Microarray Core Facility at The University of Texas Southwestern Medical Center at Dallas (http://microarray.swmed.edu) using Human Genome U133 Plus2.0 (Affymetrix GeneChip, Affymetrix, Santa Clara, CA, USA) and hybridization values were obtained for each spot by using GenePix software (Molecular Devices). The mean intensity for each gene was obtained in duplicate from each of the replicated experiments and a compound mean of signal intensity was calculated from four independent spots. The results were cutoff twofold change.

### Mass spectrometry

The HEK 293 cells transfected with flag-tagged DAB2IP were lysed and immunoprecipitated with anti-flag antibody. The protein complex mixtures were run into the SDS-PAGE gel and stained with Coomassie Blue. Identification of proteins in bands cut from Coomassie-stained SDS-PAGE gel was performed by Orbitrap Elite mass-spectrometry platforms (Thermo Fisher Scientific, Waltham, MA, USA), using short reverse-phase LC-MS/MS method. Proteins were identified from samples using our in-house data analysis pipeline (CPFP) of proteomics core at The University of Texas Southwestern Medical Center at Dallas.

### Xenograft animal model

All experimental procedures have been approved by the Institutional Animal Care and Use Committee. Six- to 8-week-old, nude, male mice were used to determine the *in vivo* tumor take rate and growth in the pre-castrated hosts. Cells (5 × 10^6^) were suspended in 200 *μ*l serum-free medium containing Matrigel (v/v, 1 : 1; BD Biosciences) and injected subcutaneously into both flanks of mice that had been castrated for 3 days. Tumor volumes were measured weekly for 8 weeks. Fresh tumor tissues were fixed in 4% paraformaldehyde for IHC analysis.

### Knockout animal model and *in situ* apoptosis detection by TUNEL staining

DAB2IP wt (DAB2IP^+/+^) and knockout (DAB2IP^−/−^) mice were described previously^15^ and 8- to 10-week-old male mice were used in this study. All mice were subjected to castration for 1, 3 and 5 days,^38^ and then killed to collect prostate. Fresh prostate tissues were fixed in 4% paraformaldehyde and paraffin-embedded sections of samples were studied by TUNEL assay or IHC staining. TUNEL staining was carried out according to the protocol provided by the manufacturer (Roche). Apoptosis was evaluated by counting the positive cells as well as the total number of cells at 10 arbitrarily selected fields at × 400 magnification in a blinded manner and quantified as the number of apoptotic cells × 100/total number of cells.

### IHC and scoring system

Mouse prostate tissues and clinical specimens were stained with antibodies specific for DAB2IP, p-STAT3 (Y705) and survivin using Dako Autostainer Plus system (Dako, Carpinteria, CA, USA) as described.^11^ The expression of DAB2IP, p-STAT3 (Y705) and survivin was scored based on the percentage and intensity according to Allred's scoring protocol.^39^ Values on a four-point scale were assigned to each specimen. The intensity score was assigned, which represented the average intensity of positive cells (0, none; 1, weak or questionably present stain; 2, intermediate intensity in a minority of cells; and 3, strong intensity in a majority of cells). All slides were scored independently by two investigators who were blinded to patient clinical information.

### Bioinformatics and statistical analyses

Two published microarray data sets were downloaded from the NCBI Gene Expression Omnibus (GEO). Obtained GEO identifiers (tissue type and sample size) were GSE4084 (PDX, *n*=52) and GSE17951 (human PCa, *n*=154). All error bars in graphical data represent mean±S.E.M. Student's *t*-test was used for the determination of statistical relevance between groups. For clinical specimen analysis, Pearson's correlation or Fisher's *Z* transform were employed. *P*<0.05 was considered significant.

## Figures and Tables

**Table 1:** Subcutaneous tumorigenesis of DAB2IP-overexpressing C4-2 or DAB2IP-knockdown LAPC-4 cells after pre-castration condition

	**No. of tumor-bearing mice (%)**	
	**4 Weeks**	**8 Weeks**
*C4-2*		
Neo	5/9 (55.6)	8/9 (88.9)
D2	0/9 (0)	0/9 (0)
		
*LAPC-4*		
Con	1/9 (11.1)	2/9 (22.2)
KD	4/9 (44.4)	7/9 (77.8)

PCa sublines (5 × 10^6^) were subcutaneously injected into pre-castrated nude mice and then the number of tumor-bearing mice was detected after 4 or 8 weeks

## Abbreviations

PCa: prostate cancer
ADT: androgen deprivation therapy
CRPC: castration-resistant PCa
AR: androgen receptor
STAT3: signal transducer and activator of transcription 3
