# Supplementary material for: DAB2IP loss confers the resistance of prostate cancer to androgen deprivation therapy through activating STAT3 and inhibiting apoptosis
Source: Cell Death Dis. 2015 Oct 29;6(10):e1955–. doi: 10.1038/cddis.2015.289 (PMC5399177; doi:10.1038/cddis.2015.289)
Supplement: Supplementary Information [file cddis2015289x1.doc]

**Supplementary Data**

**Materials and Methods**

**Cell Culture**

PZ-HPV-7 sublines were maintained in PrEGM medium (Lonza, Walkersville, MD) supplemented with 100 ng/ml Puromycin (Invitrogen, Carlsbad, CA). RWPE-1 sublines were maintained in Keratinocyte medium (Invitrogen) supplemented with 10% fetal bovine serum (FBS, Invitrogen) and 100 ng/ml Puromycin. PC-3 sublines were maintained in T-Medium (Invitrogen) supplemented with 10% FBS and 100 ng/ml Puromycin. All cells were mycoplasma-free and maintained at 37°C with 5% CO_2_ in a humidified incubator.

**Supplementary Figure Legends**

**Supplementary Fig. 1 DAB2IP modulates colony formation of PCa cells under androgen deprivation.** C4-2 (A) and LAPC-4 (B) sublines were cultured in phenol-free RPMI1640 + 5% charcoal stripped FBS, and cell growth was determined by Colony formation assay, representative pictures of colony formation in two sublines were shown.

**Supplementary Fig. 2 Endogenous DAB2IP interacts with STAT3 in prostate cells.** Cell lysates from RWPE-1 and LAPC-4 cells were subjected to co-immunoprecipitation probed with DAB2IP and then probed with t-STAT3 antibody.

**Supplementary Fig. 3 DAB2IP differently regulates STAT3 phosphorylation, survivin and Bcl-2 expression in PCa cells.** Cell lysates from PC-3 or immortalized human normal prostate epithelial PZ-HPV-7 and RWPE-1 sublines were subjected to Western blot for determining p-STAT3 (Y705), p-STAT3 (S727), t-STAT3, survivin, Bcl-2, Bax, Bcl-xL and Mcl-1 expression levels. Actin was used as a loading control. No survivin expression was detectable in immortalized normal prostate epithelial PZ-HPV-7 and RWPE-1 cells.

**Supplementary Fig. 4 Elevated expression of p-STAT3 (Y705) and survivin in DAB2IP-deficient LAPC-4 KD xenograft tissues.** LAPC-4 Con and LAPC-4 KD subcutaneous xenograft tissues from nude mice were harvested for IHC staining with p-STAT3 (Y705) and survivin antibodies, respectively. The scale bar represents 50 μm.

**Supplementary Fig. 5 DAB2IP modulates the transcription activity of STAT3 in PCa cells.** LNCaP, DU145 and PC-3 cells were transiently transfected with pLucTKS3 or its control pLucTK and DAB2IP cDNA or DAB2IP siRNA with or without 10 ng/ml IL-6 treatment for 48 hrs before luciferase assay. Each result was performed in triplicate. *p < 0.05.

**Supplementary Fig. 6 Overexpression of survivin or constitutively active STAT3C could abolish the effects of DAB2IP on the induction of cell apoptosis in C4-2 D2 cells under the ADT condition.** C4-2 D2 cells were transiently transfected with survivin cDNA or STAT3C plasmids for 36 hrs, and then cultured in phenol-free RPMI1640 + 5% charcoal stripped FBS for another 36 hrs. Cell lysates were subjected to Western blot for determining p-STAT3 (Y705), DAB2IP, survivin, cleaved PARP and cleaved Caspase-3 levels. Actin was used as a loading control.

**Supplementary Fig. 7-8 Profile of survivin and p-STAT3 expression in human PCa tissues.** The IHC staining of survivin and p-STAT3 (Y705) were performed in three different TMAs, and the analytical histogram of survivin and p-STAT3 protein expression was shown. *p < 0.05.

**Supplementary Fig. 9 DAB2IP differently regulates p53 expression in prostate cells.** Cell lysates from LAPC-4, C4-2, PZ-HPV-7 and RWPE-1 sublines were subjected to Western blot for determining p53 and DAB2IP expression levels. Actin was used as a loading control.

**Supplementary Table. 1 DAB2IP regulates gene expression profiles in PCa cells.**

**Supplementary Table. 2 Mass spectrometry showing protein profiles interacting with different domains of DAB2IP in 293 cells.**
