# Supplementary material for: DAB2IP loss confers the resistance of prostate cancer to androgen deprivation therapy through activating STAT3 and inhibiting apoptosis
Source: Cell Death Dis. 2015 Oct 29;6(10):e1955–. doi: 10.1038/cddis.2015.289 (PMC5399177; doi:10.1038/cddis.2015.289)
Supplement: Supplementary Figures [file cddis2015289x2.ppt]

## Slide 1
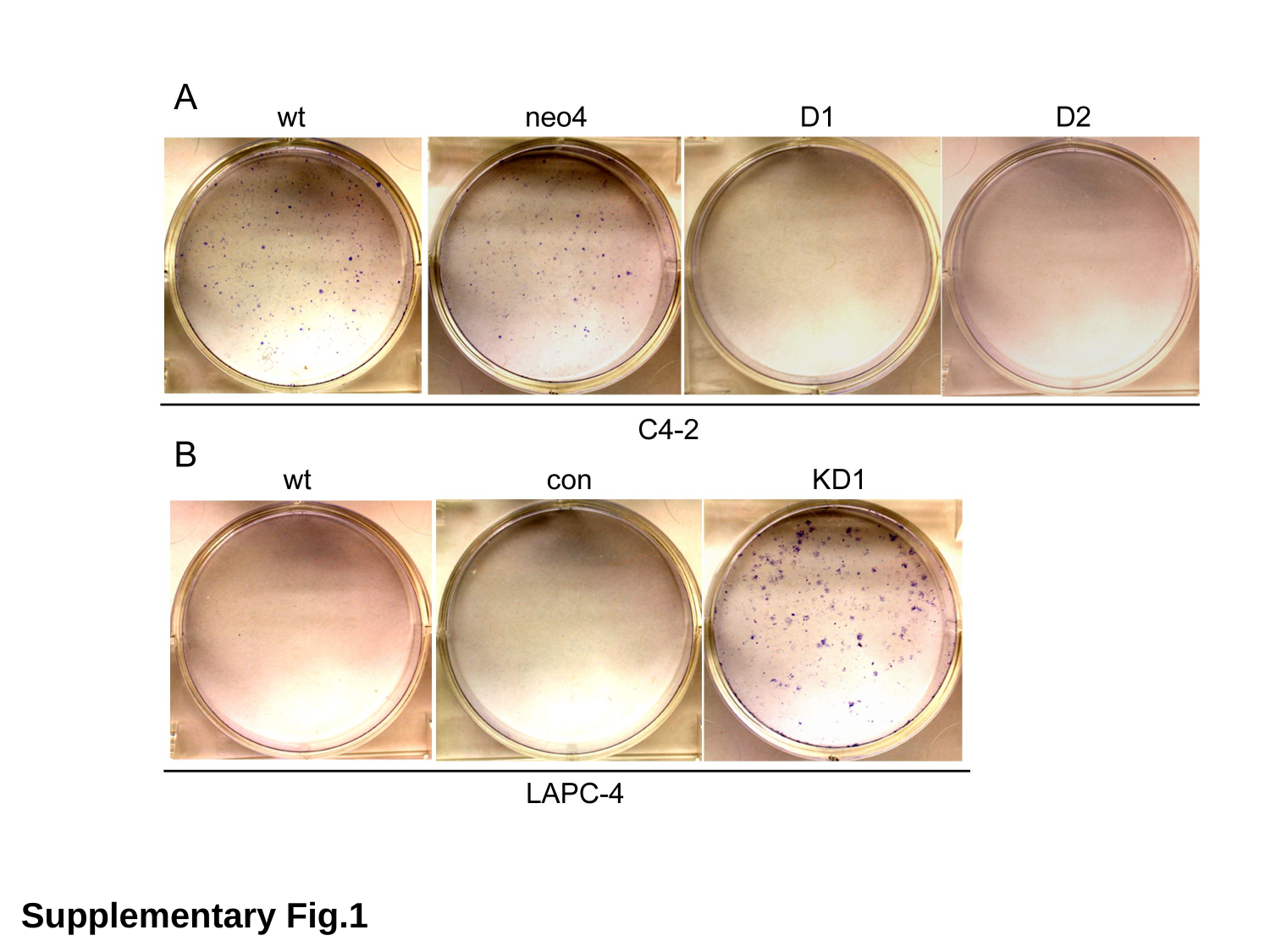

Supplementary Fig.1

## Slide 2
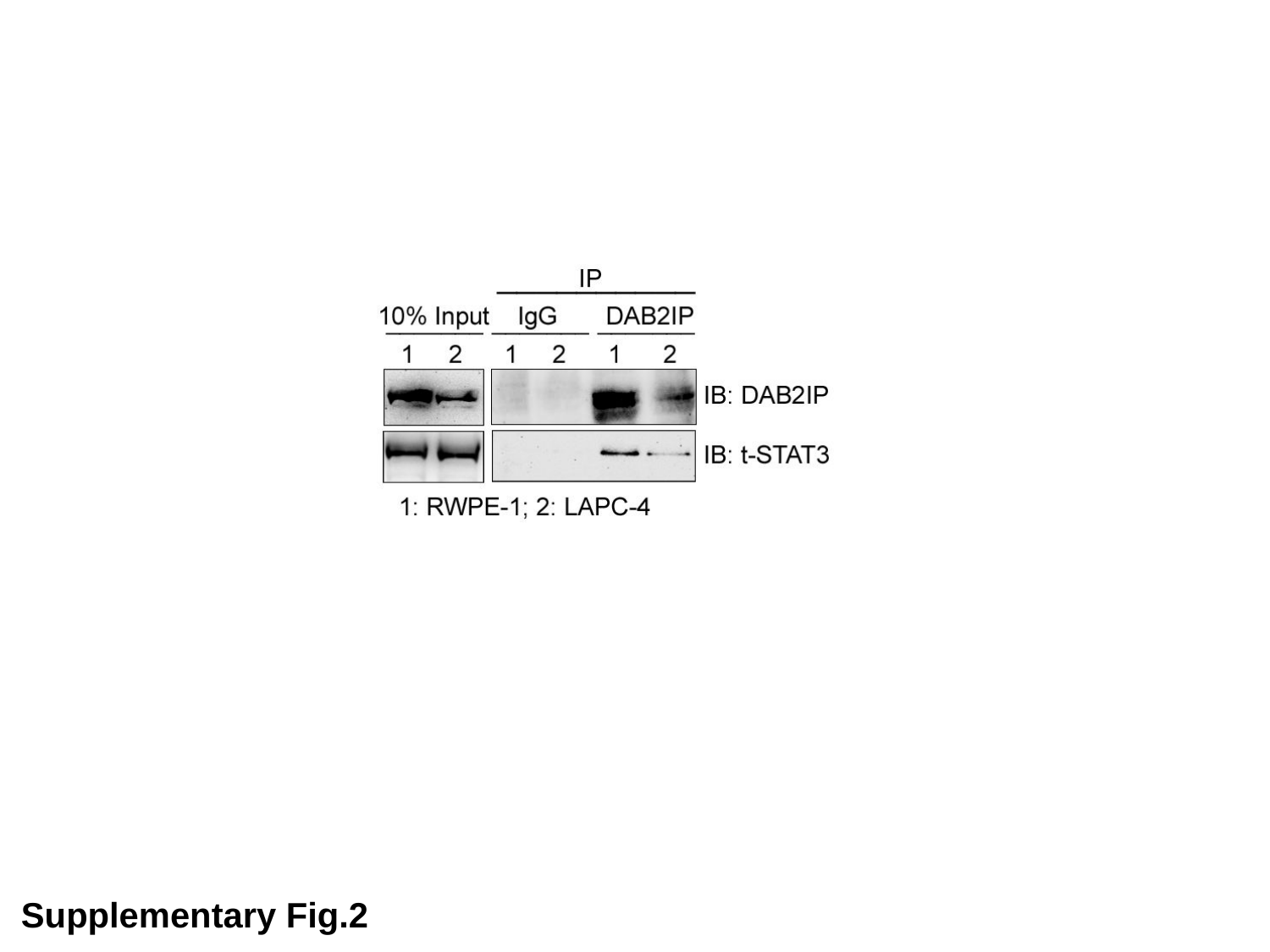

Supplementary Fig.2

## Slide 3
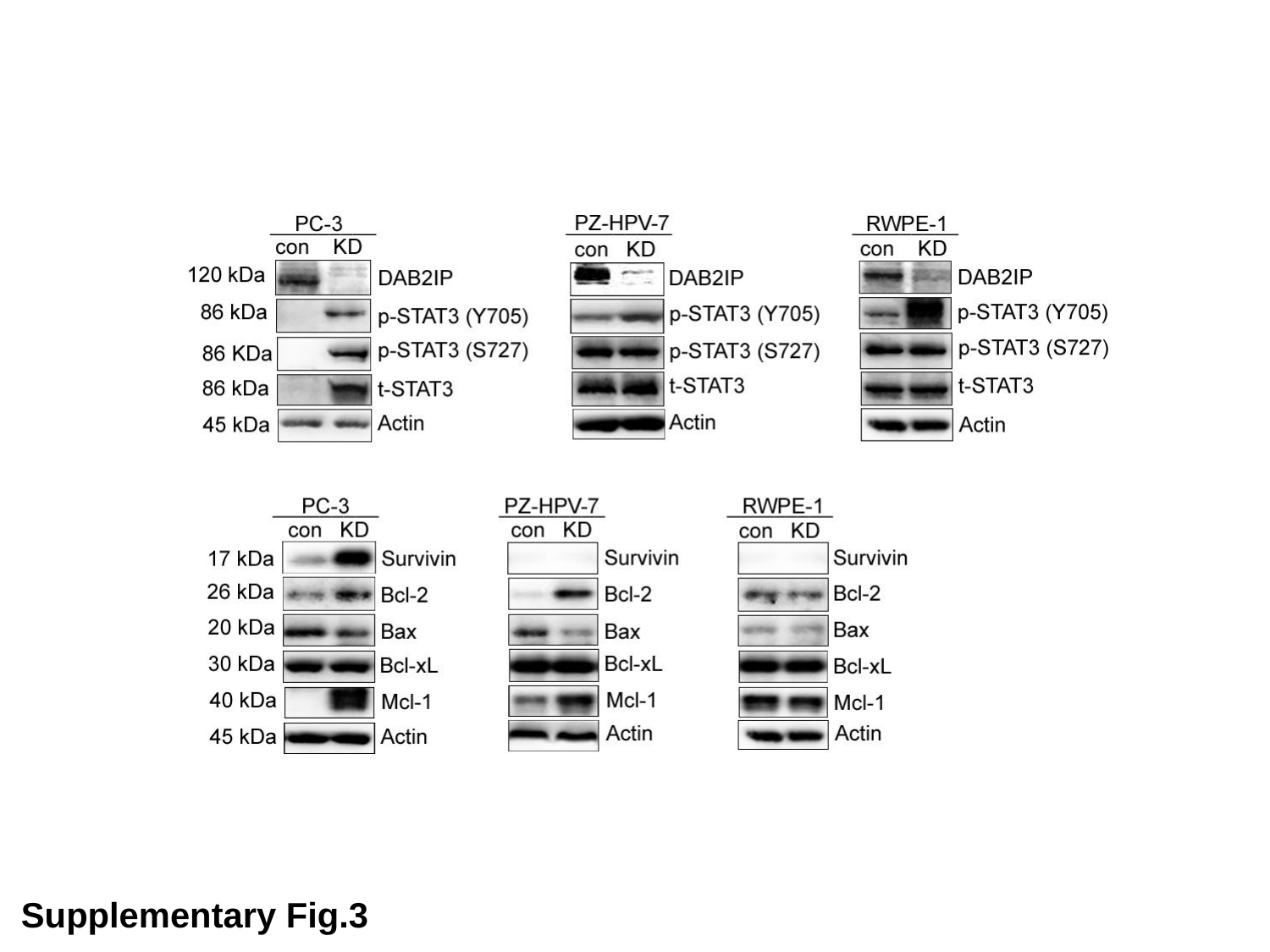

Supplementary Fig.3

## Slide 4
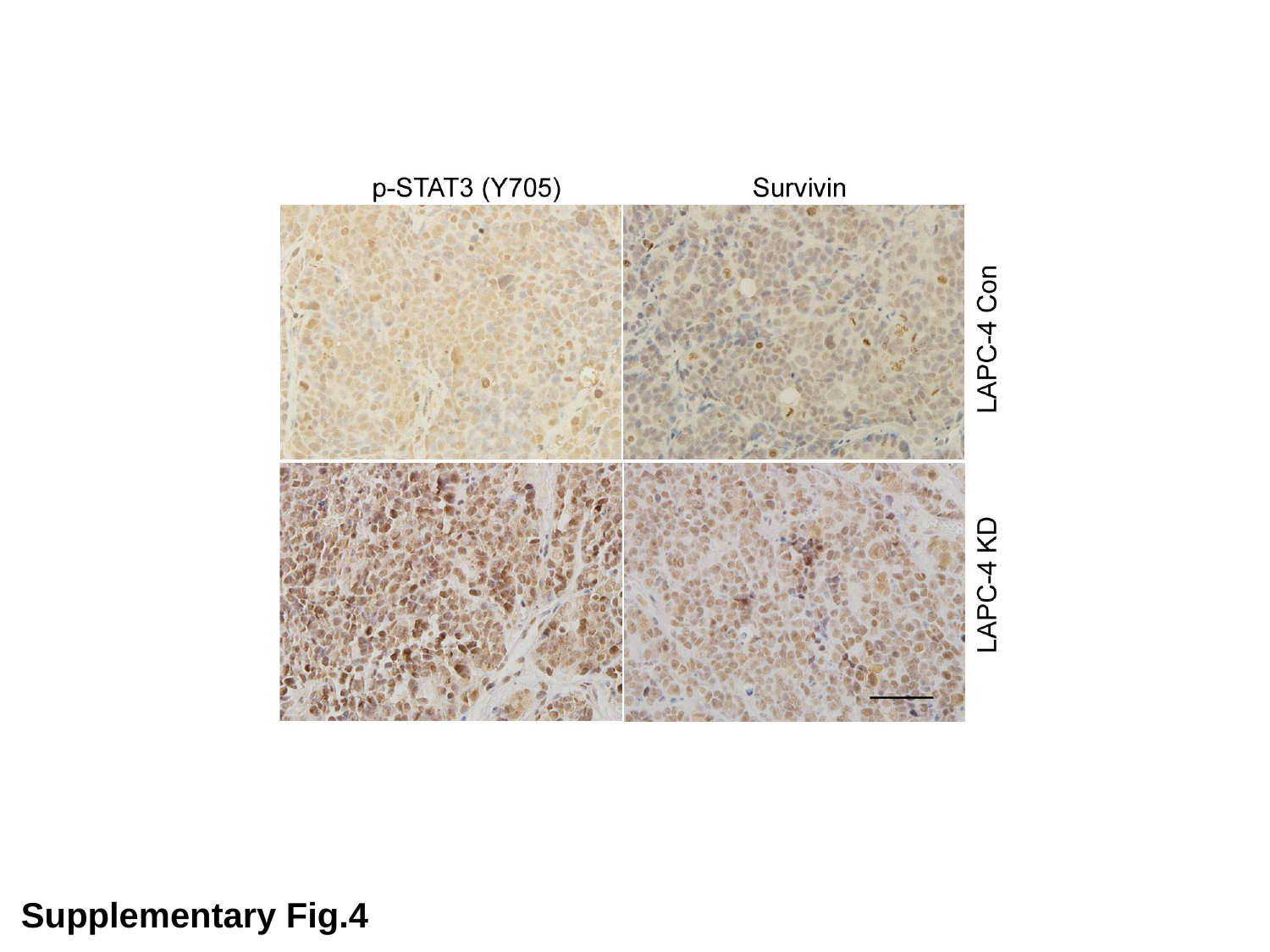

——
Supplementary Fig.4

## Slide 5
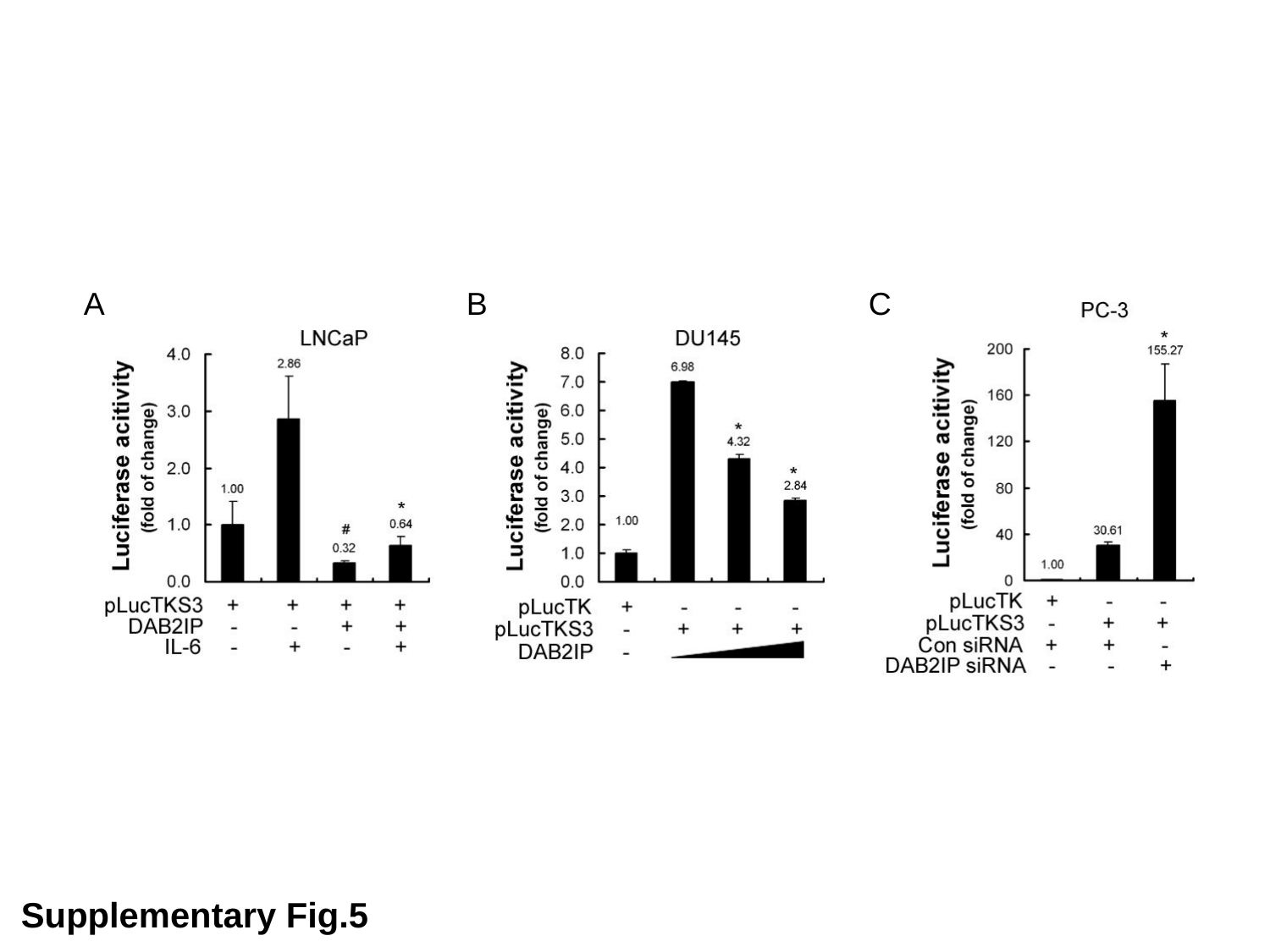

A B C
Supplementary Fig.5

## Slide 6
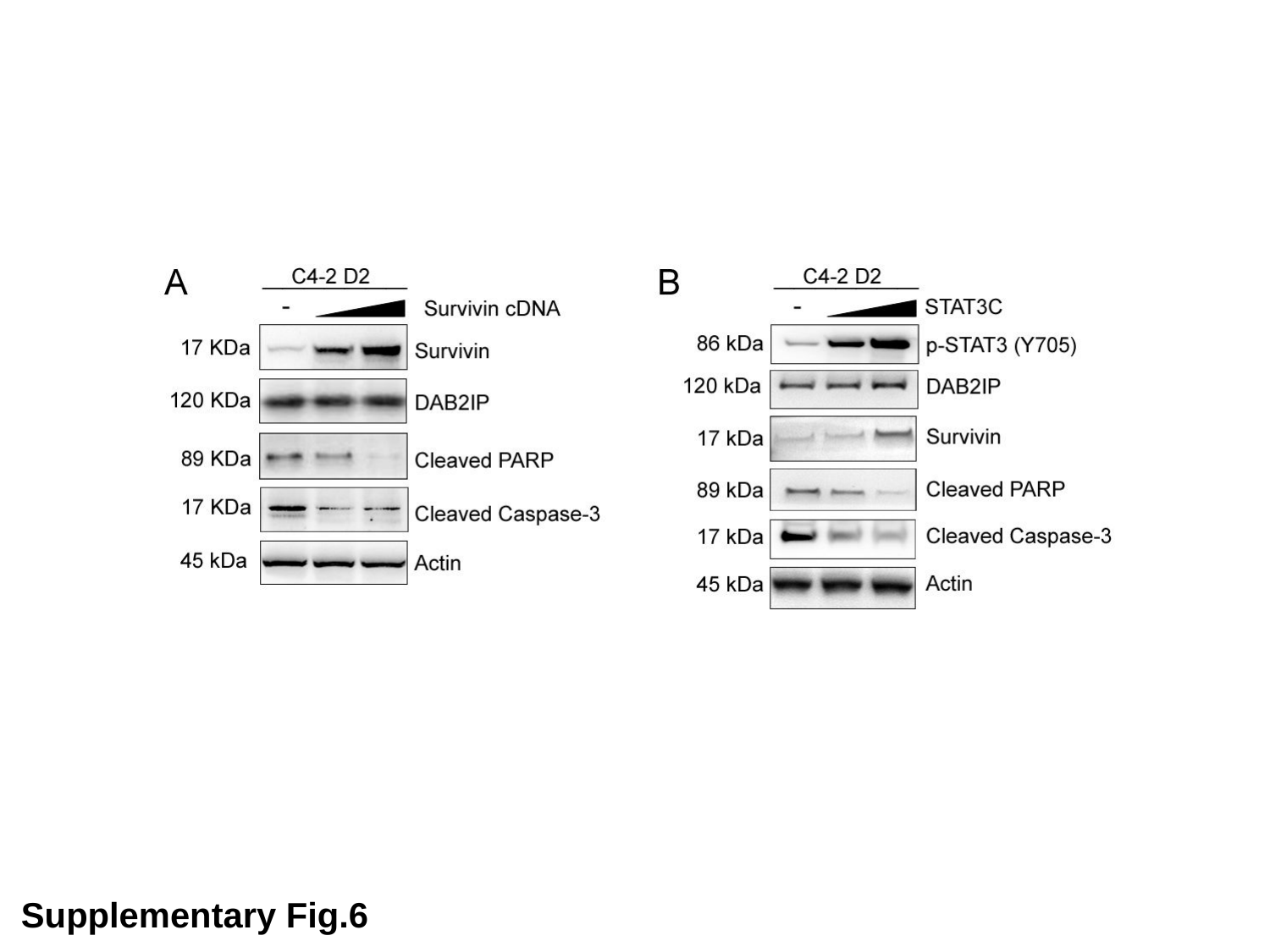

Supplementary Fig.6

## Slide 7
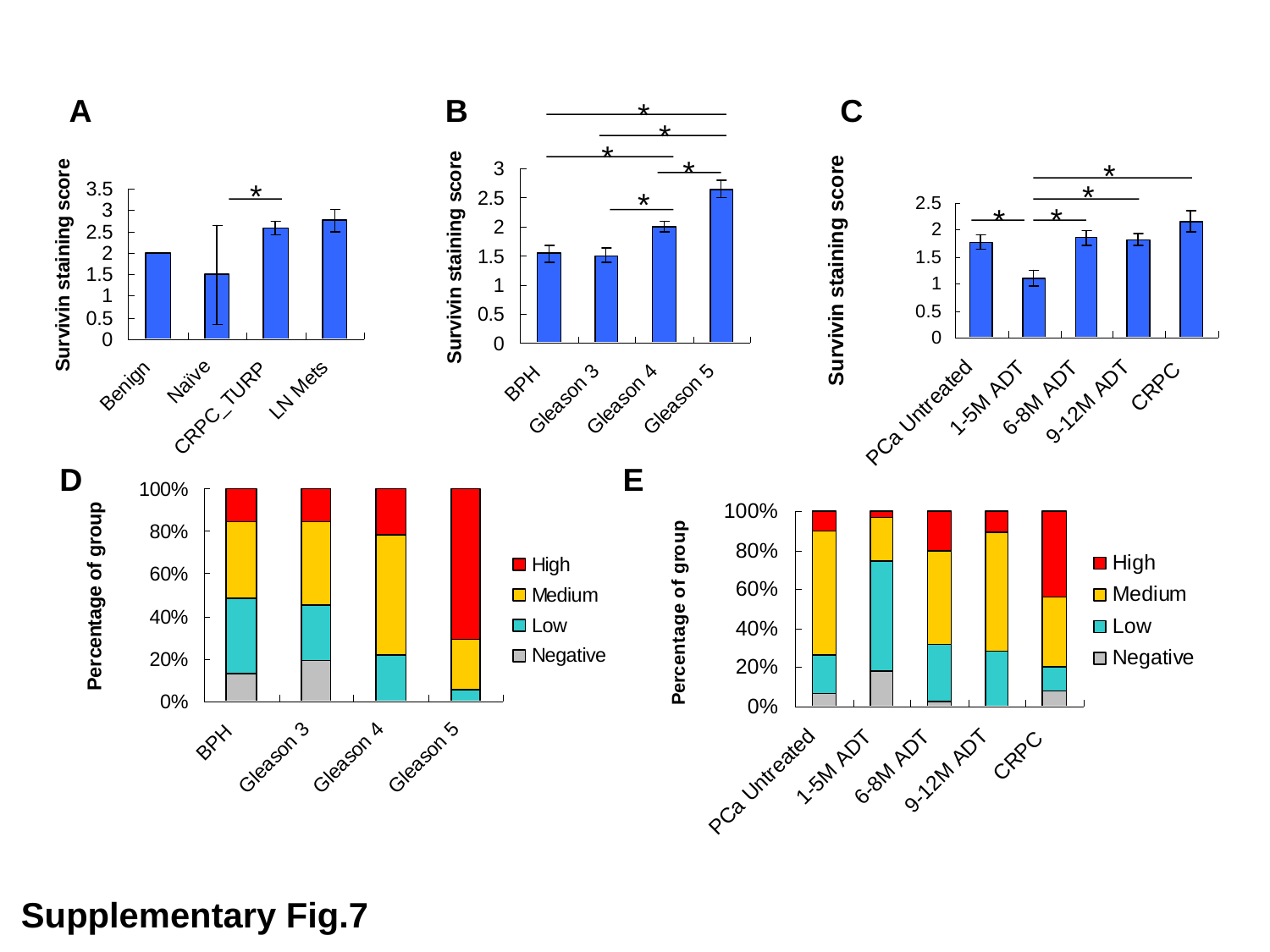

A B C
*
*
*
*
*
*
*
*
*
*
D E
Supplementary Fig.7

## Slide 8
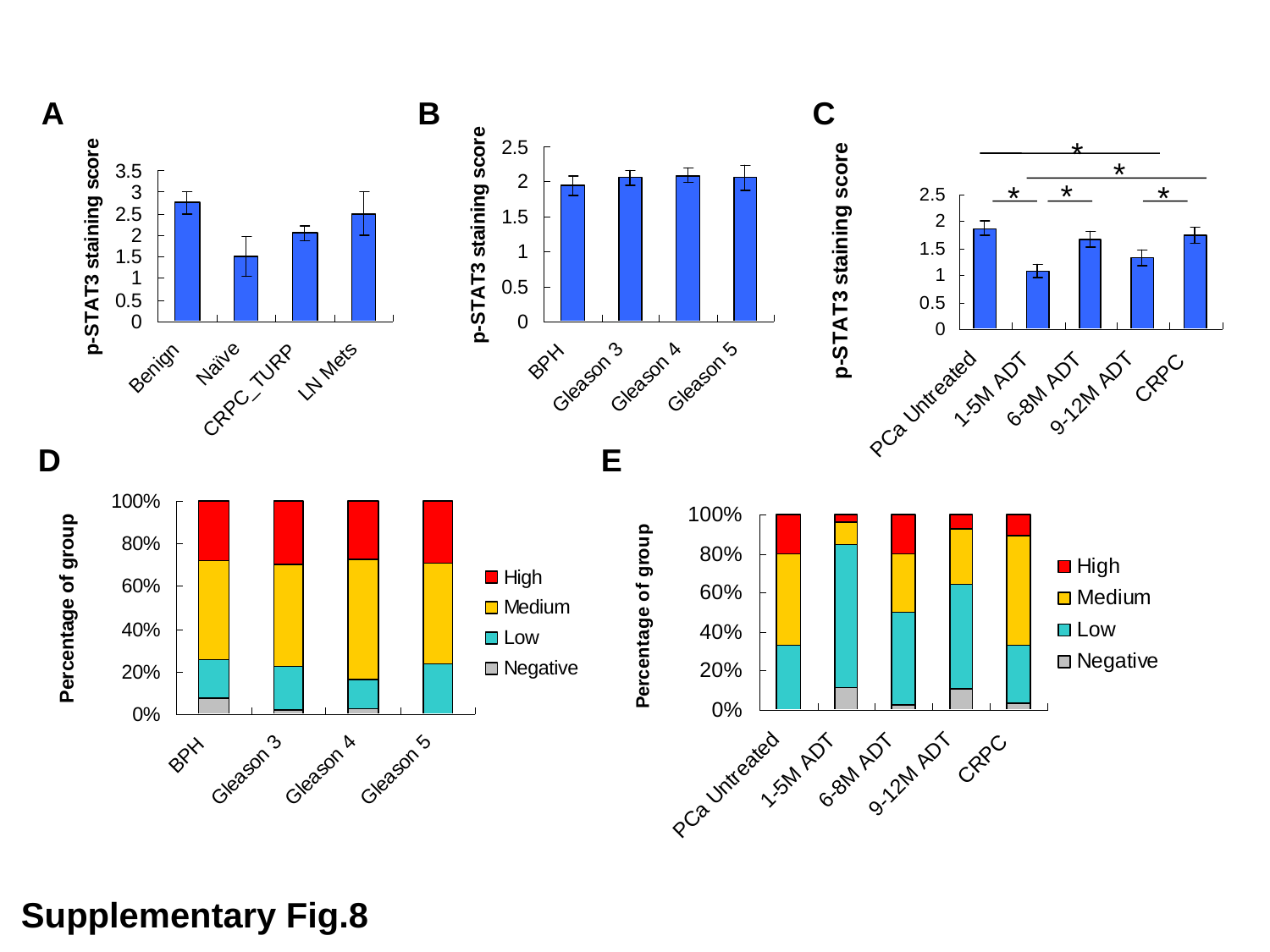

A B C
*
*
*
*
*
D E
Supplementary Fig.8

## Slide 9
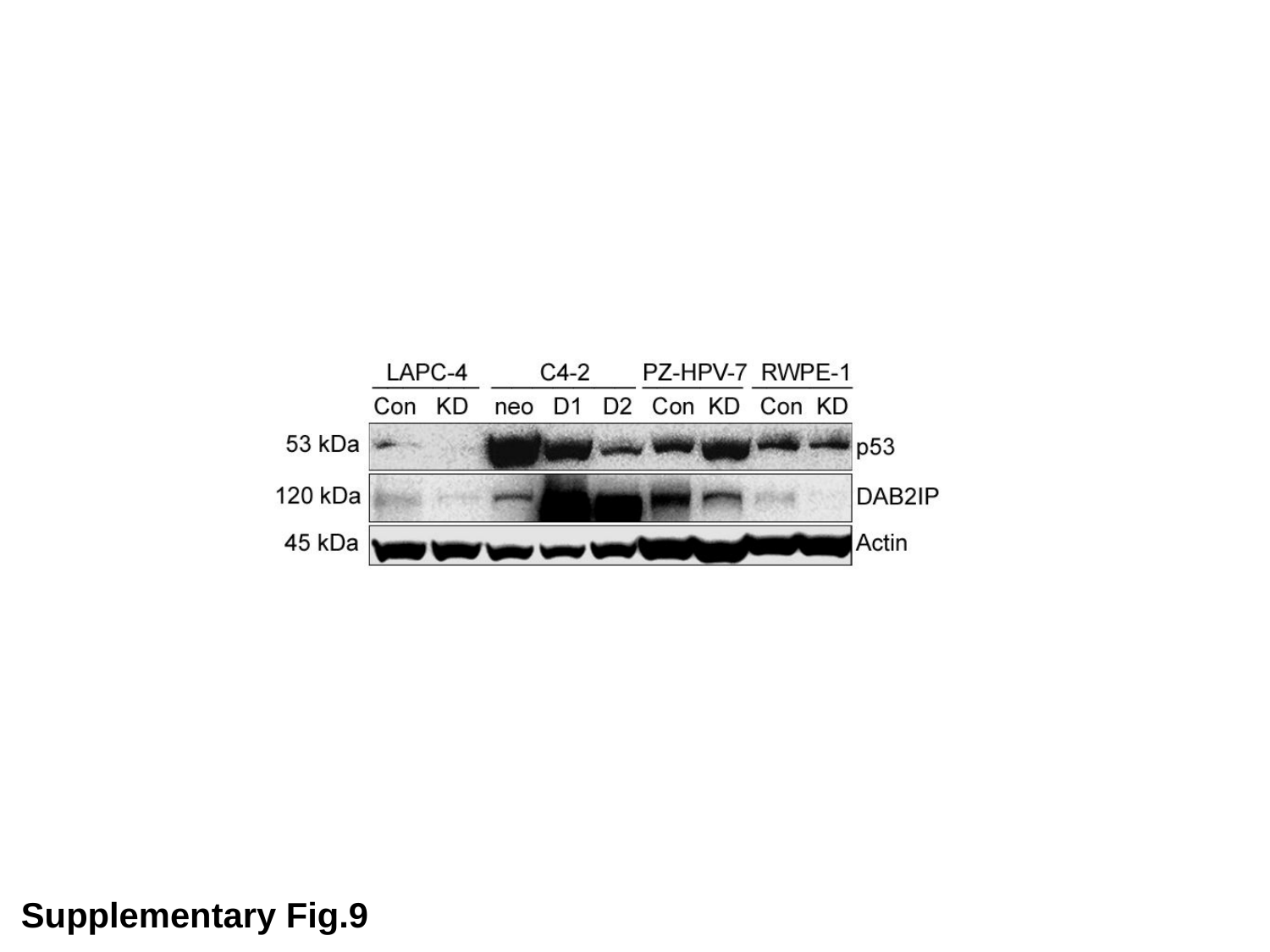

Supplementary Fig.9
